# Patient-reported GP health assessments rather than individual cardiovascular risk burden are associated with the engagement in lifestyle changes: population-based survey in South Australia

**DOI:** 10.1186/s12875-019-1066-9

**Published:** 2019-12-13

**Authors:** David Alejandro Gonzalez-Chica, Jacqueline Bowden, Caroline Miller, Marie Longo, Mark Nelson, Christopher Reid, Nigel Stocks

**Affiliations:** 1 Discipline of General Practice, Adelaide Medical School, The University of Adelaide, Helen Mayo North, Sturt Road, Level 1, Room 113. South Australia, Adelaide, 5005 Australia; 2 Adelaide Rural Clinical School, The University of Adelaide, Adelaide, SA Australia; 3 Population Health Research Group, South Australian Health & Medical Research Institute, Adelaide, SA Australia; 4 Population Health and Clinical Monitoring, Drug Policy & Population Health, Drug & Alcohol Services South Australia, Adelaide, SA Australia; 5 Menzies Institute for Medical Research, University of Tasmania, Hobart, Tasmania Australia; 6 School of Public Health, Curtin University, Perth, Western Australia Australia

**Keywords:** Cardiovascular disease, Primary prevention, Secondary prevention, Lifestyle risk reduction

## Abstract

**Background:**

Little is known about whether a more comprehensive health assessment, performed by a general practitioner (GP) during a clinical encounter, could influence patients’ lifestyle. We aimed to investigate whether health assessments, performed by GPs, are more important than the presence of cardiovascular disease (CVD) or cardiometabolic risk factors (obesity, diabetes, hypertension, dyslipidaemia) for engagement in lifestyle change.

**Methods:**

Cross-sectional, population-based survey conducted in South Australia (September–December 2017) using face-to-face interviews and self-reported data of 2977 individuals aged 15+ years. The main outcome was engagement in four lifestyle changes: 1) increasing fruit/vegetable intake, 2) increasing physical activity level, 3) reducing alcohol consumption, and 4) attempts to stop smoking. Health assessments performed by a GP in the last 12 months included clinical/laboratory investigations (weight/waist circumference, blood pressure, glucose levels, lipid levels) and questions about lifestyle/wellbeing (current diet, physical activity, smoking status, alcohol intake, mental health, sleeping problems). Results were restricted to individuals aged 35+ years because of the low prevalence of CVD or their risk factors among younger participants. Logistic regression was used in all associations, adjusted for sociodemographic, lifestyle, mental health, and clinical variables.

**Results:**

Of the 2384 investigated adults (mean age 57.3 ± 13.9 years; 51.9% females), 10.2% had CVD and 49.1% at least one cardiometabolic risk factor. Clinical/laboratory assessments performed by the GP were 2–3 times more frequent than assessments of lifestyle, mental health status, or sleeping problems, especially among those with CVD. Individuals with CVD or a cardiometabolic risk factor were no more likely to be increasing their fruit/vegetable consumption (33.6%), physical activity level (40.9%), reducing alcohol consumption (31.1%), or trying to quit smoking (34.0%) than ‘healthy’ participants. However, lifestyle changes were between 30 and 100% more likely when GPs performed three or more health assessments (either clinical/laboratory or questions about lifestyle/wellbeing) than when individuals did not visit the GP or when GPs performed no any assessment during these clinical encounters (*p* < 0.05 in all cases).

**Conclusion:**

More frequent and comprehensive CVD-related assessments by GPs were more important in promoting a healthier lifestyle than the presence of CVD or cardiometabolic risk factors by themselves.

## Background

Cardiovascular disease (CVD) carries a substantial public health burden, accounting for 36% of all deaths (40,000 deaths/year in Australia and 18 million worldwide) and 6.9% of all disability [1, 2]. In 2015–16, CVD was responsible for 8.9% ($10.4 billion) of the health expenditure in Australia [3]. Guidelines recommend a healthy diet, adequate physical activity, reduced alcohol consumption, and smoking cessation for primary and secondary CVD prevention [1, 4–6]. These recommendations have proven effective in reducing the risk of acute events, total mortality, and improving quality of life [1, 6, 7]. Nonetheless, according to studies conducted in different countries, the prevalence of an unhealthy lifestyle among those with CVD or metabolic risk factors (obesity, hypertension, diabetes mellitus, dyslipidaemia) has been found to be similar to that of individuals without them [8–10].

Despite individual factors and organisational barriers that affect the adoption of guideline recommendations [4, 7, 11–14], interventions undertaken in primary care can be effective in promoting lifestyle changes and reducing CVD risk [1, 15–17]. In fact, primary health care physicians play a central role in the prevention and management of CVD and its risk factors [1, 4, 7, 15, 16]. Lifestyle advice provided by health professionals during standard consultations have been found to improve patients behaviour, such as a 5–10% reduction of fat intake, 36% reduction of excessive alcohol consumption, increase of fruit, vegetable and fish intake, and an increase of 44–77% in the odds of quitting smoking [18–20].

Depending on the CVD risk of their patients, it is recommended that physicians perform clinical checks every 3–24 months (i.e. blood pressure measurement, weight/waist circumference checks, lipids and glucose level assessments), provide lifestyle counselling, consider medication needs/adherence, and investigate factors associated with poor management (i.e. socioeconomic context, mental health problems, co-morbidities, sleep apnoea) [1, 4–6]. Comprehensive and regular health assessments may therefore be important in improving lifestyle risk factors or reducing CVD risk [1, 6, 15, 21].

In Australia, studies in this field have mostly focused on CVD risk screening and management gaps in general practice, such as the time spent with the patient during the consultation, medication adherence, or the achievement of recommended physiological parameters [22–24]. However, little is known about whether a more comprehensive health assessment performed by a general practitioner (GP) during these encounters could influence patients’ lifestyle behaviours.

Therefore, we aimed to explore whether the presence of CVD, its cardiometabolic risk factors or recommended health assessments performed by a GP is a more important determinant for engagement in lifestyle changes.

## Methods

A cross-sectional representative face-to-face survey (Health Omnibus Survey) was conducted in South Australia (SA) by Harrison’s Research between September–December 2017. A multistage non-replacement sampling process was used to select a random sample of all individuals aged 15+ years in the state. Details of the methods have been reported previously [25, 26]. Individuals with a terminal illness or a mental incapacity (*n* = 60) or unable to speak English (*n* = 77) were ineligible for the study. Of the 4320 eligible participants, 1343 refused to answer the survey, providing a final sample of 2977 individuals (68.9%). Considering the low prevalence of CVD or their risk factors among younger participants [1], only those aged 35+ years were included in our analyses (*n* = 2384).

### Outcome: engagement in lifestyle changes

Current engagement in lifestyle changes was assessed through four different questions [1) *Are you currently increasing your consumption of fruit and vegetables?; 2) Are you currently increasing your level of exercise?; 3) Are you currently reducing your alcohol consumption?; 4) How many serious attempts have you made to quit smoking in the last year?*]. Each of the first three questions allowed the options *“No, but I have done so in the past”;* “*No, and I do not intend to in the next 6 months”; “No, but I am thinking about doing it in the next 6 months”; “No, but I intend to in the next 30 days”; “Yes, and I have been for less than 6 months”; “Yes, and I have been for more than 6 months”*. Binary variables when then created based on these answers. Participants were considered as “positives” for increasing fruit and vegetable intake or increasing physical activity level when they answered “yes” to the correspondent questions, either it was happening for less or more than six months. This cut-off was defined considering the “action” and “maintenance” stages of change defined by the Transtheoretical Model [27], which is consistent with the follow-up time used in previous studies investigating lifestyle change [15–17]. Participants were classified as reducing their alcohol consumption when they answered “yes” to the correspondent question (either for less or more than six months) and individuals self-reported drinking alcohol in the last 12 months.

For smoking cessation, individuals who reported having smoked in the last 12 months were considered as “positives” for this lifestyle change when at least one attempt to quit smoking occurred in the last year.

### Independent variables: cardiovascular conditions, risk factors and health assessments

The occurrence of CVD and its cardiometabolic risk factors was based on self-reported medical diagnosis (“*Has a doctor ever told you that you have …*” ), and included a list of CVD and their risk factors (i.e. myocardial infarction, angina, heart failure, stroke, high blood pressure, dyslipidaemia, diabetes mellitus), with the answering option “yes/no” for each of them. Body mass index (BMI), another risk factor for CVD, was defined based on self-reported weight and height and used to classify individuals as obese when the BMI was ≥30.0 kg/m^2^ [5]. All these binary variables were combined to create a new one categorized as 1) none of them (considered as ‘healthy’ in this paper); 2) at risk of CVD (with obesity, high blood pressure, dyslipidaemia, and/or diabetes, but not a diagnosed CVD), or; 3) with current or past CVD (regardless whether they had or did not have a cardiometabolic risk factor).

Participants were also asked if they had visited a GP in the last 12 months for any reason. They were then questioned about recommended preventive care/health assessments [1] performed by the GP during these visits, including 1) measurement of their weight and/or waist circumference, 2) a blood pressure check, 3) tested/requested a test to check their glucose levels, 4) tested/requested a test to check their lipid levels, 5) discussion or assessment of their diet or 6) physical activity levels, or 7) smoking status, or 8) alcohol intake, or 9) mental health status (diagnosed with anxiety, depression or other mental health problem), or 10) any sleeping problem or snoring. Each of these assessments was investigated separately as binary questions (yes/no). Moreover, they were all combined for analysis into a discrete variable (number of these health assessments performed by the GP, ranging from 0 to 10) and then transformed into an ordinal variable (0/did not visit the GP, 1–2, 3–5, 6–10 health assessments).

### Confounding variables

Sociodemographic variables [1, 7, 8, 15, 28] included sex, age, marital status, residence area, attained educational level, working status, and the Socio-Economic Indexes for Areas Index of Relative Socio-economic Advantage and Disadvantage (SEIFA-IRSAD, an indicator of relative economic and social advantage/disadvantage of people and households within an area) [29].

Current lifestyle characteristics were investigated using separate questions for fruit intake, vegetable intake, physical activity, alcohol consumption and smoking [see Additional file 1]. Additionally, mental health status (*“currently receiving treatment for anxiety, depression, or any other health problem”*) was included as a possible confounder, considering it could affect answers related to lifestyle changes and assessments performed by the GP [4, 7].

Participants were also questioned about the number of times they visited a GP, any hospitalisations, or visits to an emergency department in the last 3 months [1, 4, 7].

### Data analysis

All analyses were performed using STATA 15.1 (StataCorp, Texas, USA) and the results weighted to the inverse probability of the individual’s selection within the household and re-weighted to the estimated population in SA in 2016 [25, 26]. For reducing alcohol consumption or smoking cessation, analyses were restricted to those individuals that self-reported drinking alcohol (*n* = 1881) or smoking (*n* = 409) in the last 12 months.

Logistic regression was used in all analysis considering the binary nature of the outcomes (i.e. increasing fruit and vegetable intake, increasing physical activity level, reducing alcohol consumption, tried to quit smoking; all coded as yes/no), with adjustment for all sociodemographic variables and mental health status.

To test if the presence of CVD or cardiometabolic risk factors were associated with the engagement in lifestyle changes, results were additionally adjusted for current lifestyle (total portions of fruit/vegetable per day, days of physical activity, doses of alcohol/day, and cigarettes smoked/day), the number of visits to the GP, visits to the emergency room, and hospitalisations in the last 3 months [1, 7, 8, 15, 28]. To test the association between the number of health assessments performed by GPs and the engagement in lifestyle changes, results were further adjusted for the presence of CVD or their risk factors (no CVD, at risk of CVD, current or past CVD).

Maximum likelihood estimates (pseudolikelihood log values) for the full models were obtained, and Wald tests for heterogeneity or trend used to estimate the *p*-values due to the use of clustered weighted data. Results from all analyses were expressed as predicted adjusted prevalence instead of odds ratio to minimize confusion when interpreting study results, as many policy makers, clinicians, and researchers are not familiar with these measures of association [30].

The variance inflation factor (VIF) was investigated as an indicator of over-adjustment and collinearity between the explanatory variables. Furthermore, sex, age, educational level, the clinical health status (either with CVD or their risk factors), and the number of visits to the GP were investigated as possible effect modifiers of the relationship between the number of health assessments performed by GPs and the engagement in lifestyle changes. The interaction was tested by including in the final adjusted logistic regression models a multiplicative term between each of these variables and the number of assessments performed by the GP [31].

### Ethics

Participants provided verbal rather than written informed consent, due to the practicalities of carrying out a large-scale study and the low-risk nature of the survey content. Written parental or guardian consent was obtained for participants aged 15–17 years. All procedures performed in this study were approved by the University of Adelaide Human Research Ethics Committee (project H-097-2010).

## Results

The sample included a total of 2384 participants aged 35+ years (mean age 57.3 ± 13.9 years; 51.9% females). Table 1 shows most participants were married (72.7%), living in urban areas (72.8%), had higher than secondary educational level (66.6%) or were employed full or part-time (51.3%).

**Table 1:** Self-reported engagement in lifestyle changes and association^1^ with sociodemographic variables among individuals ≥35 years in South Australia in 2017 (*N* = 2384)

	%	Increasing fruit and vegetable intake	Increasing physical activity level	Reducing alcohol consumption^a^	Tried to quit smoking^b^
Overall (95% CI)		33.6% (31.2–36.1)	40.9% (38.4–43.4)	31.1% (28.3–33.9)	34.0% (29.1–39.3)
Sex		**		*	
Male	48.1	29.8	38.7	33.9	38.2
Female	51.9	37.2	43.0	28.0	30.0
Age group		**	***		
35–49 years	32.7	38.0	42.9	31.4	34.0
50–64 years	35.3	36.8	44.7	32.2	29.3
65–79 years	25.2	27.4	38.3	31.3	53.5
≥ 80 years	6.8	17.6	18.7	18.9	58.4
Marital status					
Married	72.7	33.0	40.2	30.4	37.4
Unmarried	27.4	35.7	42.4	33.1	31.0
Residence area				***	
Urban	72.8	33.5	40.8	27.9	35.9
Rural	27.2	34.1	41.1	40.1	32.4
Educational level		*	*		
Bachelor or higher	25.4	31.8	42.8	29.1	22.3
Trade qualification	13.1	31.5	35.5	32.4	35.9
Certificate/diploma	27.1	36.1	45.3	32.3	45.2
Secondary	22.3	37.9	39.1	33.0	27.5
Less than secondary	12.2	25.6	35.4	26.3	36.5
Working status					
Employed full time	32.9	35.8	44.4	34.4	31.0
Employed part time	18.4	36.8	37.0	29.9	39.8
Not working	16.6	29.8	38.4	32.8	43.1
Retired	32.1	31.1	40.9	26.5	24.6
Dwelling			**		
Owner	76.7	34.6	40.5	30.7	34.4
Rented privately	15.3	31.7	47.4	30.5	37.0
Government/community housing	8.0	28.0	31.1	36.7	31.6
Socioeconomic position					
Highest	18.7	35.4	36.3	35.8	23.5
High	22.0	33.9	40.0	26.7	37.1
Middle	19.0	31.7	43.5	30.1	31.1
Low	16.4	29.0	38.9	29.9	39.9
Lowest	23.9	37.0	45.2	32.9	34.8

*P*-value * < 0.05; ** < 0.01; *** < 0.001

^1^ Results considering mutual adjustment between all sociodemographic variables and mental health status. Maximum likelihood estimates (pseudolikelihood log) values for the full models: increasing fruit and vegetable intake = − 1257.6; increasing physical activity level = − 1339.7; reducing alcohol consumption = − 983.5; tried to quit smoking = − 223.5

^a^ Results for the 1881 individuals that consumed alcohol in the last 12 months

^b^ Results for the 409 individuals that smoked in the last 12 months

Table 1 also shows engagement in lifestyle changes and their association with sociodemographic variables. The proportion of participants currently increasing their fruit and vegetable intake (33.6%) was lower in females, elderly individuals, or among those with less than secondary educational level. The proportion of individuals increasing their physical activity level (40.9%) was also less frequent in the oldest age or less educated groups, or among those living in Government/community housing. Among those who consumed alcohol in the last 12 months (*n* = 1881; 50.2% males), women were less likely than men to be reducing their alcohol consumption (28.0 vs 33.9), as well as those living in urban areas. Among those who were smoking in the last 12 months (*n* = 409; 17.2% of the sample), 40.7% tried to quit smoking in the last year, but no significant association was found with any sociodemographic variable.

Table 2 summarises the prevalence of CVD and its risk factors and their relationship with engagement in lifestyle changes. Hypertension was the most frequent risk factor (35.8%) and myocardial infarction or angina the most frequent CVD (9.7%). Overall, 93.7% of all participants in the sample had visited a GP in the last 12 months (95% CI 92.4–94.8) and this percentage was almost 100% among those at risk or with CVD. Two-thirds of the sample showed inadequate fruit/vegetable intake or physical activity levels, while 28.6% consumed more than two standard doses of alcohol per day and 16.1% were current smokers. Neither individual at risk nor with CVD showed better compliance with any lifestyle recommendation. In fact, those with obesity, dyslipidaemia, diabetes or myocardial infarction/angina showed a higher prevalence of inadequate physical activity levels.

**Table 2:** Self-reported prevalence of cardiovascular diseases or their cardiometabolic risk factors and association with the inadequacy of lifestyle recommendations among individuals ≥35 years in South Australia in 2017 (*N* = 2384)

	%	Visited GP (%)^a^	Inadequacy of lifestyle recommendations^1^			
			Low fruit/ vegetable intake (%)^b^	Low physical activity level (%)^b^	High alcohol consumption (%)^b^	Current smoker (%)
Overall (95%CI)		93.7 (92.4–94.8)	64.8 (62.5–67.0)	67.1 (64.7;69.4)	28.6 (26.4–30.9)	16.1 (14.2–18.3)
Cardiometabolic risk factors						
Obesity^c^	26.1	97.6**	66.7	75.3***	30.7	13.1
Hypertension	35.8	98.7***	67.5	70.0	28.9	14.6
Dyslipidaemia	30.2	98.3***	61.2	70.9*	28.5	16.7
Diabetes mellitus	14.9	99.3**	61.7	74.1*	25.4	16.2
Cardiovascular disease						
Myocardial infarction or angina	9.7	99.1*	61.1	75.6**	28.6	16.8
Heart failure	2.7	100.0**	56.5	77.1	13.0	25.0
Stroke	2.0	96.9	64.3	69.0	17.1	8.5

*P*-value * < 0.05; ** < 0.01; *** < 0.001

^1^ Results adjusted for sociodemographic variables (sex, age, area of residence, marital status, education level, dwelling, socioeconomic position, working status) and mental health status

^a^ Visited a GP in the last 12 months for any reason

^b^ Low fruit and vegetable intake < 5 portions of fruit and/or vegetables/day; low physical activity level < 150 min/week of moderate/vigorous physical activity; high alcohol consumption > 2 standard doses of alcohol/day

^c^ Body mass index ≥30.0 kg/m^2^ based on self-reported information for weight and height

Regarding lifestyle changes (Table 3), diabetes mellitus was the only metabolic risk factor associated with some of the outcomes (reducing alcohol consumption). Individuals with heart failure were also twice as likely to have tried to quit smoking than their peers, but also less prone to have reduced their alcohol consumption. None of the other clinical conditions was associated with a more frequent engagement in any of the investigated lifestyle changes.

**Table 3:** Association between cardiovascular diseases or their cardiometabolic risk factors and engagement in lifestyle changes among individuals ≥35 years in South Australia in 2017 (*N* = 2384)

	Lifestyle changes^1^			
	Increasing fruit and vegetable intake	Increasing physical activity level	Reducing alcohol consumption^a^	Tried to quit smoking^b^
Cardiometabolic risk factors				
Obesity^c^	35.2	42.0	33.1	33.5
Hypertension	31.3	40.6	33.7	29.9
Dyslipidaemia	34.8	41.8	33.2	39.1
Diabetes mellitus	32.2	45.7	38.6*	35.9
Cardiovascular disease				
Myocardial infarction or angina	39.3	46.7	32.3	38.9
Heart failure	29.3	41.4	13.3*	66.8*
Stroke	32.9	46.0	38.8	60.3

*P*-value * < 0.05; ** < 0.01; *** < 0.001

^1^ Results adjusted for sociodemographic variables (sex, age, area of residence, marital status, education level, dwelling, socioeconomic position, working status) and mental health status, and current lifestyle characteristics (portions of fruit/vegetable per day, days of physical activity, doses of alcohol/day, and cigarettes smoked/day). ^a^ Analyses restricted to individuals that consumed alcohol in the last 12 months (*n* = 1881)

^b^ Analyses restricted to individuals that smoked in the last 12 months (*n* = 409)

^c^ Body mass index ≥30.0 kg/m^2^ based on self-reported information for weight and height

Table 4 shows that even when these clinical conditions were analysed as a combined variable (no CVD, at risk of CVD, current or past CVD), they were not associated with any of the lifestyle change variables (*p*-value > 0.05 in all cases). The same table also shows that clinical/laboratory assessments performed by GPs (especially blood pressure assessment) were 2–3 times more frequent than the assessment of lifestyle, mental health, or sleeping problems. Despite these differences, most of these assessments were more frequent among patients with CVD. The number of assessments performed by GPs (median 4 assessments; interquartile range 2–6) increased as the patient reported having a metabolic risk factor or CVD.

**Table 4:** Patient self-reported prevalence of health assessments performed by their GP in the last 12 months and engagement in lifestyle changes, stratified according to the presence of self-reported clinical condition. Individuals ≥35 years, South Australia, 2017 (*N* = 2384)

		Prevalence of clinical condition^1^			
	Overall %	None (40.7%)	Cardiometabolic risk factor^a^ (49.1%)	Cardiovascular disease^b^ (10.2%)	*p*-value*
		%	%	%	
Lifestyle change					
Increasing fruit and vegetable intake	33.6	32.4	34.3	35.9	0.361
Increasing physical activity level	40.9	40.6	40.3	45.6	0.353†
Reducing alcohol consumption^c^	31.1	28.3	32.9	34.6	0.070
Tried to quit smoking^d^	34.0	36.0	32.1	39.9	0.573
Assessments performed by the GP^e^					
Measured weight and/or waist	46.9	35.8	51.3	64.7	< 0.001
Checked blood pressure	87.9	81.8	92.6	95.5	< 0.001
Tested glycaemia	62.4	50.9	68.7	74.6	< 0.001
Tested lipid levels	66.3	54.4	72.9	79.7	< 0.001
Assessed diet	29.5	17.0	35.4	46.6	< 0.001
Assessed physical activity	32.8	23.5	36.7	45.7	< 0.001
Assessed smoking status	18.5	18.5	17.1	25.8	0.017†
Assessed alcohol intake	20.4	18.6	20.8	25.6	0.070
Assessed mental health status	27.9	25.9	28.0	36.0	0.025
Assessed sleeping problems/snoring	23.0	20.1	23.6	29.5	0.011
No. of assessments – *Median [IQR]*^*2*^	*4[2–6]*	*3.2[1.7–4.8]*	*4.3[2.8–6.1]*	*5.0[3.3–6.7]*	*< 0.001*

* Test for trend; † Test for heterogeneity; *IQR* interquartile range

1 Results adjusted for sociodemographic variables (sex, age, area of residence, marital status, education level, dwelling, socioeconomic position, working status), lifestyle variables (portions of fruit/vegetable per day, days of physical activity, doses of alcohol/day, and cigarettes smoked/day), mental health, number of visits to the GP, hospitalisations, and visits to the emergency room. Maximum likelihood estimates (pseudolikelihood log) values for the full models: increasing fruit and vegetable intake = − 1220.6; increasing physical activity level = − 1271.6; reducing alcohol consumption = − 946.0; tried to quit smoking = −203.6

2 Unadjusted results

^a^ Including individuals with body mass index ≥30 kg/m^2^, hypertension, diabetes and/or dyslipidaemia, but without cardiovascular diseases

^b^ Including heart attack, angina, heart failure, and/or stroke (with or without metabolic risk factors)

^c^ Analyses restricted to individuals that consumed alcohol in the last 12 months (*n* = 1881)

^d^ Analyses restricted to individuals that smoked in the last 12 months (*n* = 409)

^e^ Analyses restricted to the 2267 individuals that visited the GP in the last 12 months (93.7% of the sample)

**Fig. 1:**
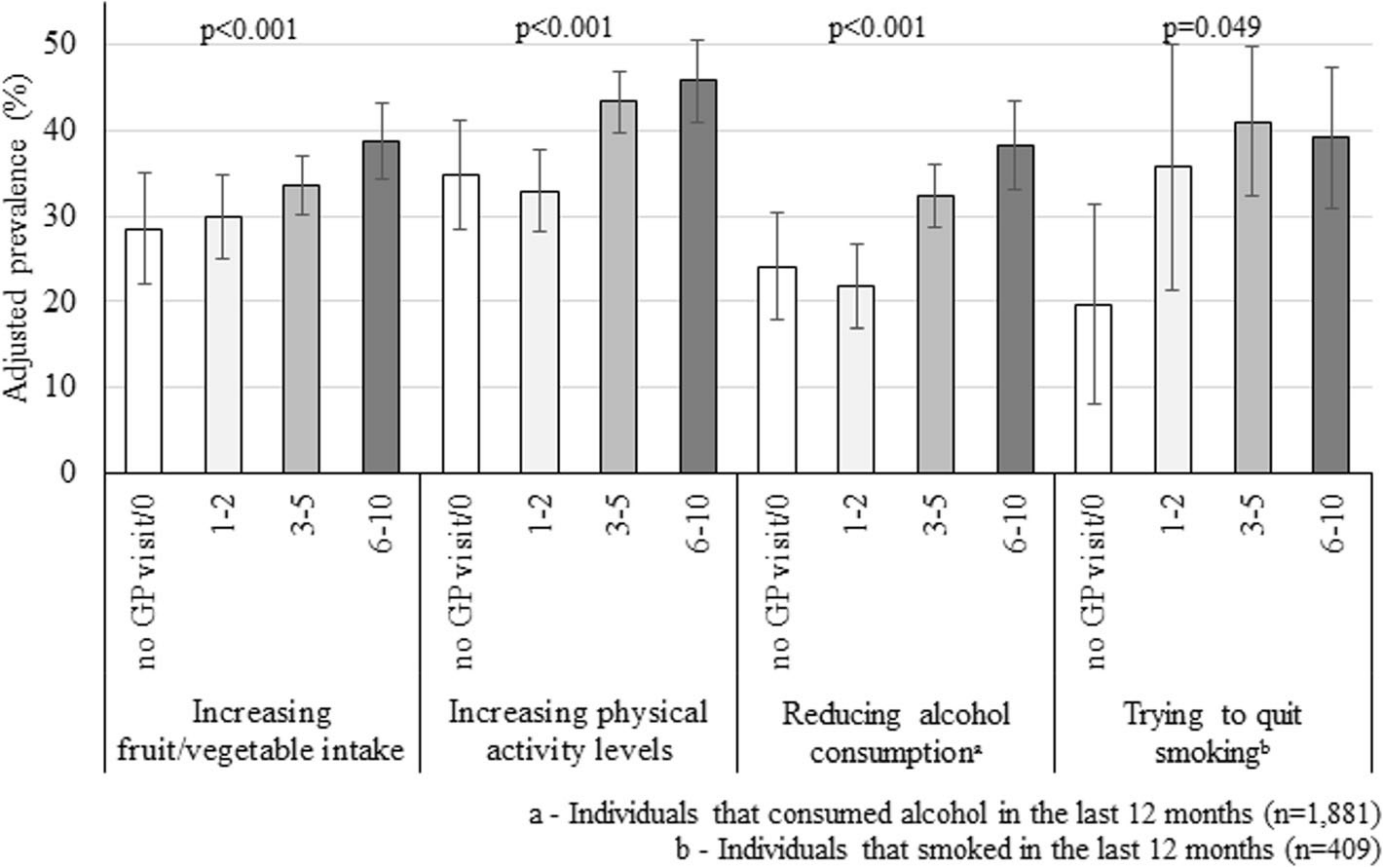
Adjusted association between the number of patient self-reported health assessments performed by their GP in the last 12 months and the engagement in lifestyle changes. Individuals ≥35 years, South Australia, 2017 (*N* = 2384). Assessments performed by the GP included the investigation of weight and/or waist, blood pressure, glycaemia, lipid levels, diet, physical activity, smoking status, alcohol intake, mental health status, sleeping habits/snoring. Results adjusted for sociodemographic variables (sex, age, area of residence, marital status, education level, dwelling, socioeconomic position, working status), lifestyle variables (portions of fruit/vegetable per day, days of physical activity, doses of alcohol/day, and cigarettes smoked/day), health status (none, at risk, or with CVD), mental health, number of visits to the GP, hospitalizations, and visits to the emergency room. Maximum likelihood estimates (pseudolikelihood log) values for the full models: increasing fruit and vegetable intake = − 1215.6; increasing physical activity level = − 1262.0; reducing alcohol consumption = − 933.0; tried to quit smoking = − 199.8. Vertical lines represent 95% CI

Figure 1 shows that all lifestyle changes were more likely among individuals whose GPs performed three or more assessments than among those who did not visit the GP or reported no assessment (*p* < 0.05 in all cases). No evidence of heterogeneity of the associations according to sex, age, educational level, clinical health status, or the number of visits to the GP were identified (interaction *p*-values > 0.10 in all cases).

The mean VIF ranged between 2.3 and 2.4 in all analyses, indicating no collinearity between the explanatory variables in the final models.

## Discussion

This large population-based survey has three main findings. First, more than 90% of adults visited their GP in the previous 12 months, especially those at risk of or with CVD. Second, individuals at risk of or with CVD reported that GPs performed more comprehensive assessments during their consultations, especially clinical/laboratory assessments. However, neither the adequacy of lifestyle recommendation nor engagement in lifestyle changes were more frequent among those at risk of or with CVD. Finally, a more comprehensive CVD-related assessment by GPs (i.e., clinical/laboratory and lifestyle/wellbeing assessments) was associated with more frequent engagement in lifestyle changes. This positive effect of health assessments performed by GPs on lifestyle changes was independent of the individual’s risk factors or CVD status.

A healthy lifestyle is widely recommended for the management of CVD and other chronic diseases, as healthy habits reduce the incidence of acute events, decrease mortality, and improve quality of life [1, 6–10]. However, studies in Australia and other high-income settings have found the prevalence of unhealthy diets (~ 65%), sedentarism (~ 60%), harmful alcohol consumption (~ 30%), or current smoking (~ 20%) among individuals at risk of or with CVD are similar to that of people without these conditions [8–10]. The prevalence observed in these studies and the lack of compliance with lifestyle recommendations among those affected by CVD or their risk factors is consistent with our findings. Failure to achieve lifestyle changes are multifactorial and involve patient-related (i.e., socioeconomic disadvantage, lack of understanding/education about the condition, reduced motivation, older age, physical limitations, depression), provider-related (i.e., communication barriers, short consultation time, health access difficulties), and environment-related factors (i.e., social pressure, long working hours) [4, 7, 11–14].

Our findings identified that when GPs performed a more comprehensive assessment during their consultations (i.e., six or more of the clinical/laboratory, lifestyle and/or wellbeing investigated aspects) patients were more likely to report lifestyle changes. Compared to those reporting no assessment or who did not visit a GP, increasing fruit and vegetable intake was 36% higher, increasing physical activity 31% more frequent, while reducing alcohol consumption and attempts to quit smoking were 59 and 98% more prevalent, respectively. These results are consistent with the current literature which shows that closer involvement of GPs and other primary health workers is essential to the uptake and maintenance of healthy behaviours, as well as adequate management of CVD and their risk factors [1, 4, 7, 15, 16, 21].

It is reassuring that those at risk of, or with CVD, who could benefit from visiting a GP are doing so in Australia, as most of them visited a doctor in the last 12 months. Moreover, around 70% of individuals with these conditions had their glycaemia and lipid levels tested, more than 90% had their blood pressure assessed, and up to 65% had their weight or waist circumference measured. However, the assessment of other factors affecting CVD risk such as lifestyle, mental health status or sleeping problems was as low as 17%. These results are consistent with the literature, as studies using direct observation of physicians during medical encounters identified smoking or physical activity was discussed in only 20% of these encounters, 10% of eligible patients received dietary advice, and the average time providing advice on these aspects was less than 1 min [15]. Therefore, according to our findings, GPs are at least partially following current guidelines for CVD risk monitoring and prevention [1, 4, 5]. These medical encounters should be used as an opportunity to assess clinical parameters and prescribe the appropriate medication for the management of CVD, but also to encourage lifestyle changes and explore other factors possible affecting wellbeing [15, 18].

In fact, health education and counselling play a central role in the 16 cost-effective, affordable, feasible and scalable strategies proposed by the World Health Organisation that would help avoiding 9.6 million premature deaths associated with CVD and other non-communicable chronic conditions by 2025 [32]. Nonetheless, further research is necessary to explore what can be realistically achieved in general practice consultations to influence patients’ lifestyle behaviours.

Some limitations should be considered in the interpretation of our results. First, the cross-sectional design of the study precludes causal inference. Moreover, there was a recall period of up to 12 months for GP assessments, and the information obtained was not confirmed by checking clinical records. However, our findings are consistent with results of longitudinal studies available in the literature and the prevalence of lifestyle assessments is similar to those from studies using direct observation during medical encounters [15–17]. Second, the engagement in lifestyle changes may be overestimated because of social desirability bias. However, participants were unaware of the objectives of this study and the question regarding health assessments and clinical health status were included at the end of the survey to minimise this source of bias. Moreover, it is unlikely social desirability affected our results, as it would have been expected a higher prevalence of lifestyle changes among those at risk of or with CVD. Finally, although we used self-reported data for the diagnosis of health conditions, these variables have been found to be highly specific [33, 34] and with a sensitivity of up to 85% [34]. In any case, misclassification and residual confounding cannot be completely excluded.

## Conclusions

This population-based study provides evidence that a more comprehensive assessment by GPs during a consultation can improve CVD prevention and encourage healthy lifestyle changes in patients. Because most adults visit a GP at least once a year, and our results apply to those with, or at risk of CVD, there is wide scope to influence patients’ intentions to adhere to lifestyle recommendation. Our research findings should encourage GPs to apply assessment of clinical risk factors/laboratory tests, education about healthy habits and contextual factors during these and other consultations because such strategies improve the lifestyle choices of patients. Further research should try to clarify if it is the frequency of visits, the content of the consultation or patient factors that have led to the associations identified in our results.

## Supplementary information

**Additional file 1.** Lifestyle questions used to create the variables related to the adequacy of lifestyle risk factors recommendations.

## Data Availability

The datasets used and/or analysed during the current study are available from the corresponding author on reasonable request.

## Abbreviations

95%CI: Confidence intervals of 95%
BMI: Body mass index
CVD: Cardiovascular disease
GP: General practitioner
p25-p75: Interquartile range
SA: South Australia
SEIFA-IRSAD: Australian Socio-Economic Indexes for Areas Index of Relative Socio-economic Advantage and Disadvantage
VIF: Variance inflation factor
