# Supplementary material for: Patient-reported GP health assessments rather than individual cardiovascular risk burden are associated with the engagement in lifestyle changes: population-based survey in South Australia
Source: BMC Fam Pract. 2019 Dec 13;20:173. doi: 10.1186/s12875-019-1066-9 (PMC6911269; doi:10.1186/s12875-019-1066-9)
Supplement: Supplementary file 1 — Additional file 1. Lifestyle questions used to create the variables related to the adequacy of lifestyle risk factors recommendations. [file 12875_2019_1066_MOESM1_ESM.docx]

**Supplementary Table S1.** Lifestyle questions used to create the variables related to the adequacy of lifestyle risk factors recommendations

| **Variables** | **Question included in survey** | **Additional information provided during the interview** |
| --- | --- | --- |
| Fruit intake | How many serves of fruits do you usually eat each day? A serve is equal to one medium piece of fruit, or two small pieces of fruit ore one cup of diced pieces.  1. Enter number of serves __ __  2. Less than 1 serve  3. None  4. Don’t know/can’t say | A prompt card with examples of a serve size was shown to the participant during the interview |
| Vegetable intake | How many serves of vegetables do you usually eat each day? A serve is equal to half a cup of cooked vegetables or one cup of salad vegetables.  1. Enter number of serves __ __  2. Less than 1 serve  3. None  4. Don’t know/can’t say | A prompt card with examples of a serve size was shown to the participant during the interview |
| Physical activity | How many days in the past week have you done any vigorous physical activity for a total of at least 30 minutes, OR any combination of moderate and/or vigorous physical activity for a total of at least 60 minutes? This can include 60 minutes of moderate exercise only.  Enter days in the last week (0-7): ____ | Additional information provided to the participant: “Vigorous activity includes activities like fast cycling, jogging, doing an exercise class, and moderate activity includes things like brisk walking, medium paced swimming, walking to and from work, gardening, doing housework etc.” |
| Alcohol consumption: frequency | In the last 12 months, how often did you have an alcoholic drink of any kind?  1. Everyday  2. 5 to 6 days a week  3. 3 to 4 days a week  4. 1 to 2 days a week  5. 2 to 3 days a month  6. About 1 day a month  7. Less often  8. Not in the last 12 months  9. No longer drink  8. Never drunk alcohol | Instruction to the interviewer: “If no longer drink ask if within the last 12 months.” |
| Alcohol consumption: quantity | On a day that you have an alcoholic drink, how many standard drinks do you usually have?  1. 20 or more drinks  2. 16-19 drinks  3. 13-15 drinks  4. 11-12 drinks  5. 9-10 drinks  6. 7-8 drinks  7. 5-6 drinks  8. 3-4 drinks  9. 2 drinks  10. 1 drink  11. Half a drink | A prompt card with examples of the equivalence of a standard drink according to the type of drink was shown to the participant during the interview |
| Smoking status: current smoking | Do you currently smoke cigarettes, cigars, pipes or any other tobacco products?  1. Daily  2. At least weekly (not daily)  3. Less often than weekly  4. Not at all |  |
| Smoking status: stopped smoking | When (date or age) did you finally stop smoking daily? | This information was allowed to be registered as an specific date, participant’s age, or the time gap since the patient stopped smoking. |
| Smoking status: quantity | On average how many manufactured cigarettes do you smoke per day (daily) or each week (weekly)?  On average how many roll your own cigarettes do you smoke per day (daily) or each week (weekly)? |  |
